# Supplementary material for: Mechanistic insights into RAN translation within the huntingtin coding sequence
Source: NAR Mol Med. 2026 Oct 22;3(4):ugag048. doi: 10.1093/narmme/ugag048 (PMC13748972; doi:10.1093/narmme/ugag048)
Supplement: Supplementary file 1 [file ugag048_supplemental_file.pdf]

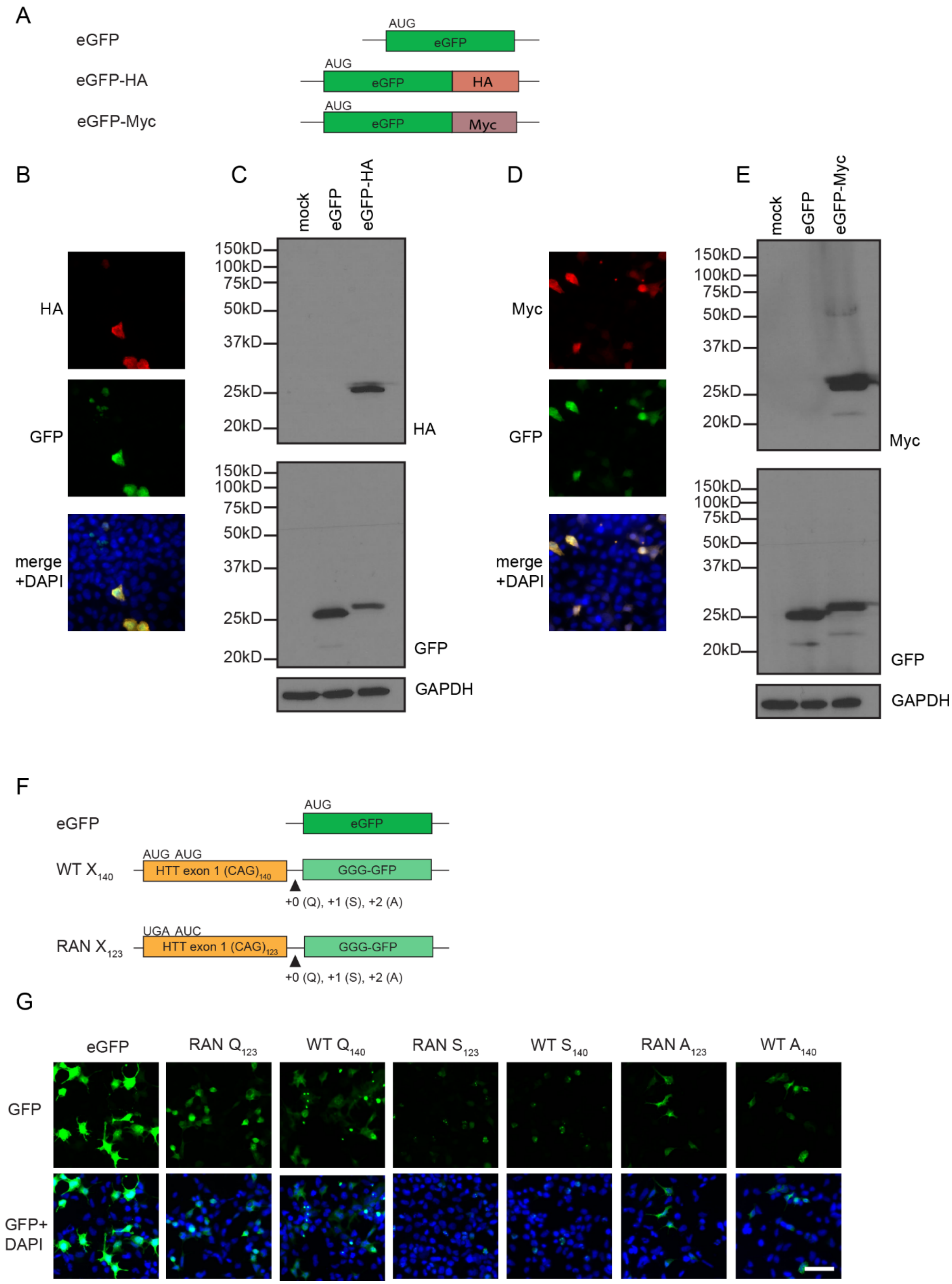
A
eGFP
AUG
eGFP
eGFP-HA
AUG
eGFP
HA
eGFP-Myc
AUG
eGFP
Myc
B
HA
GFP
merge
+DAPI
C
mock
eGFP
eGFP-HA
150kD
100kD
75kD
50kD
37kD
25kD
20kD
HA
GFP
GAPDH
D
Myc
GFP
merge
+DAPI
E
mock
eGFP
eGFP-Myc
Myc
GFP
GAPDH
F
eGFP
AUG
eGFP
WT X140
AUG AUG
HTT exon 1 (CAG)140
GGG-GFP
+0 (Q), +1 (S), +2 (A)
RAN X123
UGA AUC
HTT exon 1 (CAG)123
GGG-GFP
+0 (Q), +1 (S), +2 (A)
G
eGFP
RAN Q123
WT Q140
RAN S123
WT S140
RAN A123
WT A140
GFP
GFP+
DAPI

Supplemental Figure 1. Localization of RAN translation products from HTT reporters. A. Schematic of the eGFP control plasmid and eGFP plasmids with either a C-terminal HA or Myc tag. Representative images and western blots of HA (B-C) and Myc (D-E) in HEK293 cells transfected with eGFP with a C-terminal HA or C-terminal Myc tag. GAPDH was used as a loading control. F. Schematics of HTT plasmids with a C-terminal eGFP without an AUG start codon. G. Representative images of HEK293 cells 48hrs after transfection with the indicated GFP (G). DAPI was a counterstain for nuclei. Scale bar is 50μm.

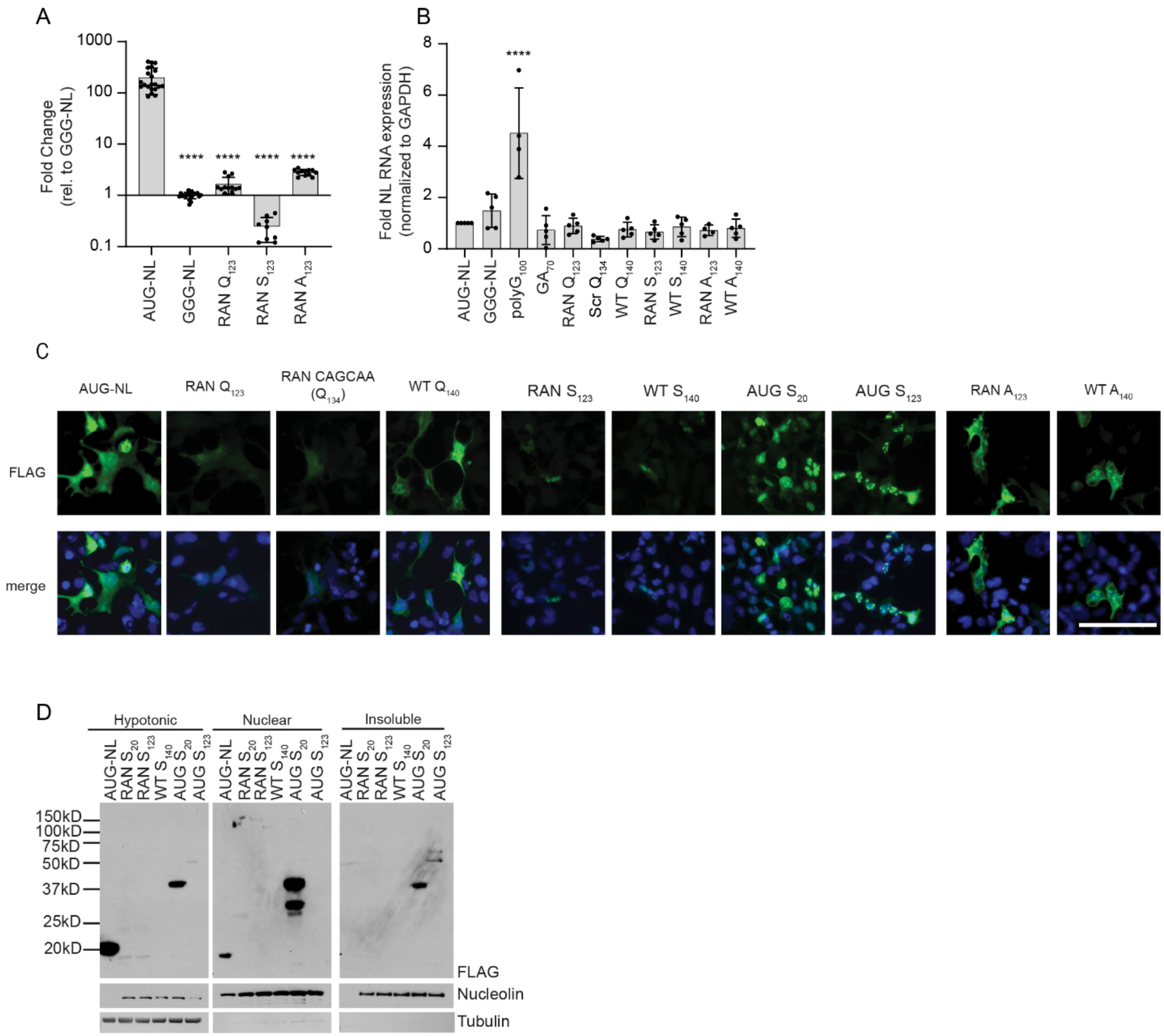

Supplemental Figure 2. RNA and protein expression from different RAN translation reporters. A. NL assay comparing luminescence of HTT RAN expressing plasmids to AUG- or GGG-NL. B. Relative mRNA expression for identified constructs in transfected HEK293 cells. GAPDH was used as a normalization control. C. Immunocytochemistry of NL reporters. D. Representative western blot of polyserine products in different cellular fractions of transfected HEK293 cells. Nucleolin was used as a nuclear marker. Tubulin was used as a cytoplasmic/hypotonic marker. Graphs are means ± SD from triplicate samples and at least 3 different experiments. A-B was an ordinary one-way ANOVA with Dunnett's correction for multiple comparisons. **** $p<0.0001$. Scale bar in C is 50μm.

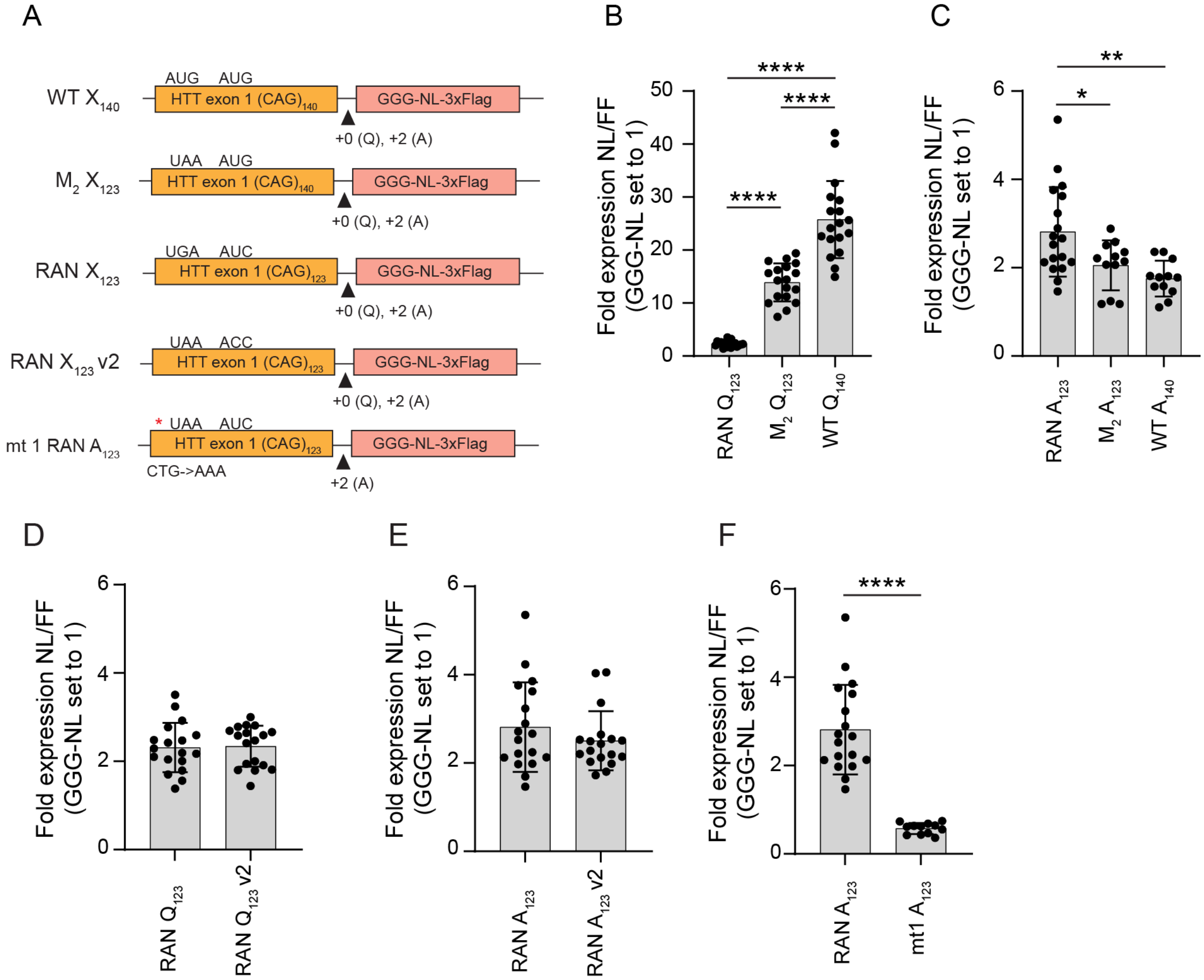

Supplemental Figure 3. Individual mutation analysis with RAN translation HTT reporter plasmids. A. Schematic of the different codon mutations made to either glutamine or alanine frame HTT plasmids. * represents the location of the mutated CUG codon. B-C. NL assay comparing the effect of the second M codon on glutamine translation or alanine translation, respectively. D-E. NL assay comparing the effect of mutating the second M to a non-near-cognate codon on the translation of the glutamine or alanine frame, respectively. F. NL assay of the effect of the near-cognate CUG codon on alanine RAN translation. Graphs are means ± SD from triplicate samples and at least 3 different experiments. B-C was an ordinary one-way ANOVA with Tukey's correction for multiple comparisons. D-F was Welch's t-test. * $p<0.05$, ** $p<0.01$, *** $p<0.0001$.

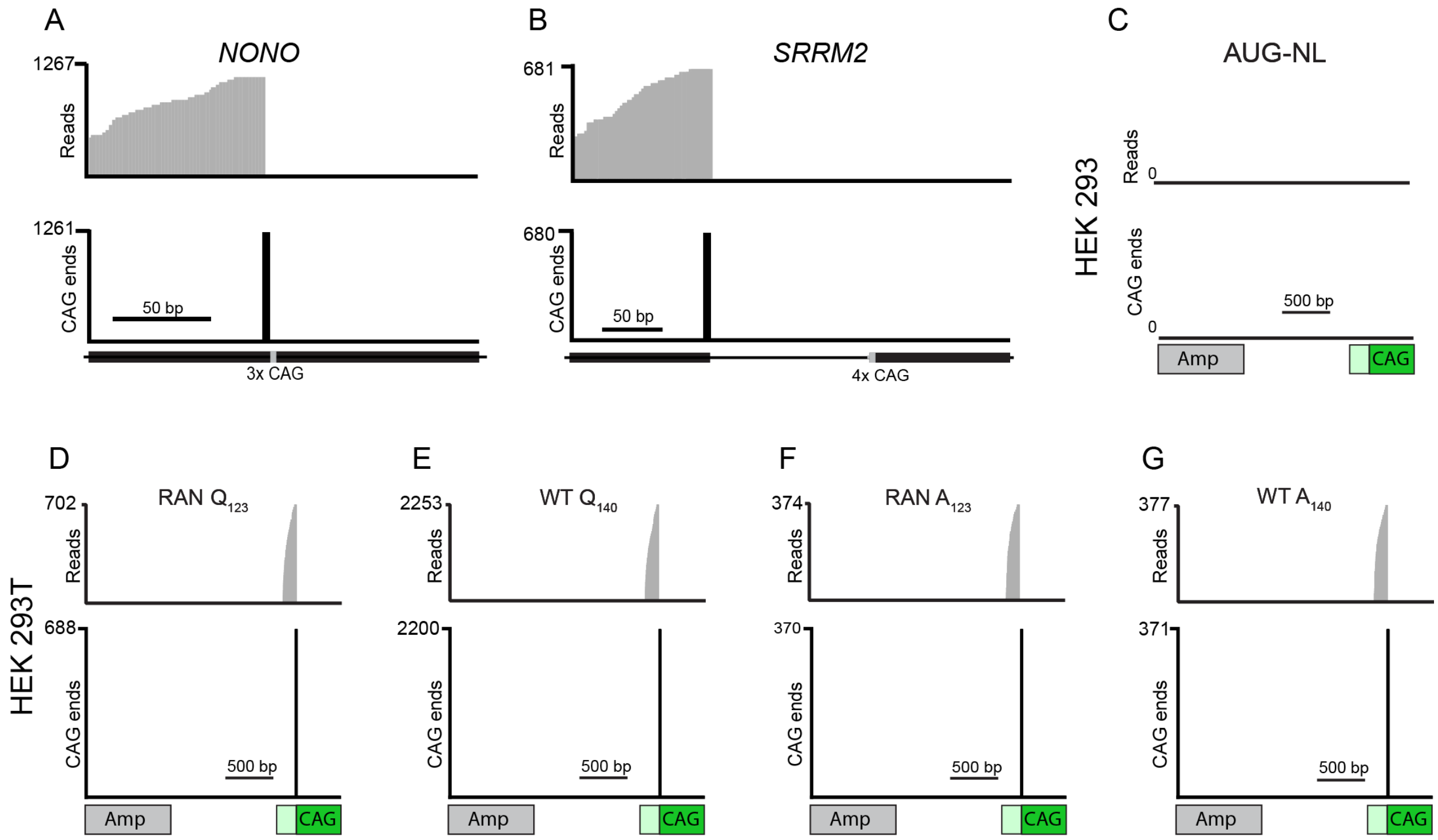

Supplemental Figure 4: Assessment for 5' ends of CAG repeat containing transcripts derived from genomic genes and from additional plasmids in HEK293 and HEK293T cells. CAG ends determined using the SATCFinder pipeline for the genes *NONO* (A) and *SRRM2* (B) for HEK293 cells transfected with the AUG-NL plasmid. CAG ends in the transfected plasmid in HEK293 cells for the AUG-NL (C). CAG ends for the transfected plasmid in HEK293T cells for the RAN Q_{123} (D), WT Q_{140} (E), RAN A_{123} (F), and WT A_{140} (G) plasmids.

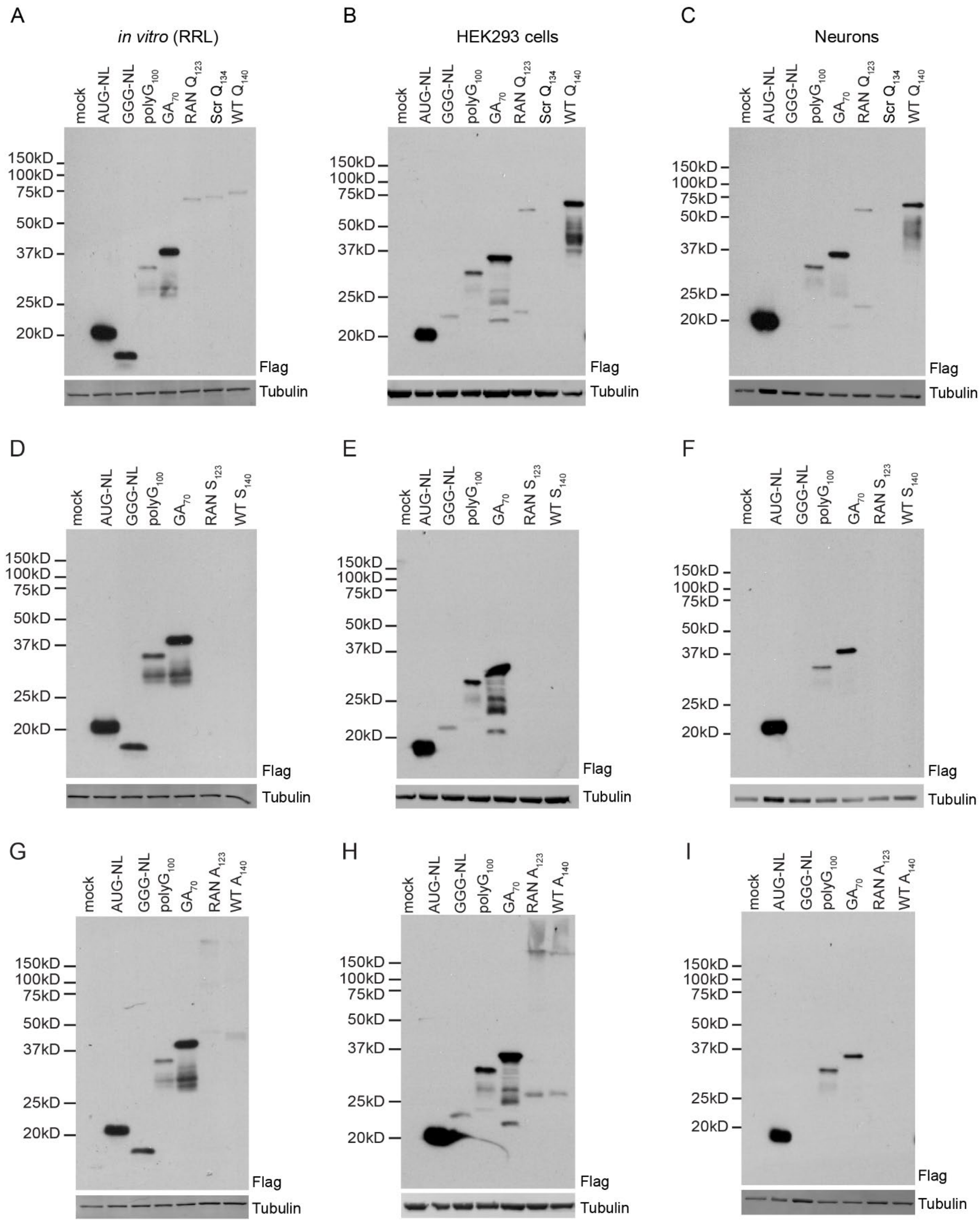

Supplemental Figure 5: RAN reporter immunoblots derived from in vitro transcribed RNA reporters. Representative western blots from IVT RNA for the glutamine frame in RRL (A), HEK293 cells (B), or primary rodent neurons (C). Representative western blots from IVT RNA for the serine frame in RRL (D), HEK293 cells (E), or primary rodent neurons (F).

Representative western blots from IVT RNA for the alanine frame in RRL (G), HEK293 cells (H), or primary rodent neurons (I). Tubulin was used as a loading control.

Supplemental Table 1: Start codons upstream of repeat-containing reads in RAN samples are due to sequencing artifact. Repeat containing reads from RAN samples containing upstream start codons identified using ATGFinder are listed below. Sample includes the cell type and transfected plasmid. Read ID refers to unique read coordinates and read mate. Each ATG was due to 1 or 2 base mismatches compared to reference and the position of these mismatches relative to repeat start is denoted in the position column. Two of these mismatches were near the read start and soft-clipped by the STAR aligner. Those not soft clipped were poor quality sequences where the chance of error was higher than the occurrence of the observed ATG sequence.

Sample	Read ID	Position	Soft Clip	Phred	Sequence Error Probability	Coverage
HEK 293 RAN A123	1290:25455:12570 R2	-52	No	12	0.0631	4378
HEK 293 RAN A123	1184:38362:29388 R2	-52	Yes	--	--	--
HEK 293 RAN Q123	1175:32900:13719 R2	-31	Yes	--	--	--
HEK293T RAN A123	2159:15069:22789 R2	-66, -65	No	12, 12	0.0631	313, 315
